# Microbiological quality and safe handling of enteral diets in a hospital in Minas Gerais, Brazil

**DOI:** 10.1590/S1517-838246220131141

**Published:** 2015-06-01

**Authors:** Raquel Oliveira Medrado Pinto, Eliznara Fernades Correia, Keyla Carvalho Pereira, Paulo de Souza Costa, Daniele Ferreira da Silva

**Affiliations:** 1 Universidade Federal dos Vales do Jequitinhonha e Mucuri, Departamento de Nutrição, Universidade Federal dos Vales do Jequitinhonha e Mucuri, Diamantina, MG, Brasil, Departamento de Nutrição, Universidade Federal dos Vales do Jequitinhonha e Mucuri, Diamantina, MG, Brazil.; 2 Universidade Federal dos Vales do Jequitinhonha e Mucuri, Instituto de Ciência e Tecnologia, Universidade Federal dos Vales do Jequitinhonha e Mucuri, Diamantina, MG, Brasil, Instituto de Ciência e Tecnologia, Universidade Federal dos Vales do Jequitinhonha e Mucuri, Diamantina, MG, Brazil.

**Keywords:** enteral diet, aseptic conditions, contamination

## Abstract

Contamination of enteral diets represents a high risk of compromising the patient's medical condition. To assess the microbiological quality and aseptic conditions in the preparation and administration of handmade and industrialized enteral diets offered in a hospital in the Valley of Jequitinhonha, MG, Brazil, we performed a microbiological analysis of 50 samples of diets and 27 samples of surfaces, utensils, and water used in the preparation of the diets. In addition, we assessed the good handling practices of enteral diets according to the requirements specified by the Brazilian legislation. Both kinds of enteral diets showed contamination by coliforms and *Pseudomonas* spp. No sample was positive for *Staphylococcus aureus* and *Salmonella* spp. On the other hand, *Listeria* spp. was detected in only one sample of handmade diets. Contamination was significantly higher in the handmade preparations (p < 0.05). Nonconformities were detected with respect to good handling practices, which may compromise the diet safety. The results indicate that the sanitary quality of the enteral diets is unsatisfactory, especially handmade diets. Contamination by *Pseudomonas* spp. is significant because it is often involved in infection episodes. With regard to aseptic practices, it was observed the need of implementing new procedures for handling enteral diets.

## Introduction

Enteral Nutrition Therapy (ENT) involves therapeutic actions intended to recover and/or maintain the patient's nutritional status satisfactorily, and is indicated for patients who are not able to receive at least two thirds of their daily energy needs orally (Waitzberg *et al.*, 2004). Usually these patients are hospitalized, or are immunosuppressive and more susceptible to infections either by microorganisms considered pathogenic or by so-called opportunistic microorganisms. Therefore, enteral feeding requires special care because it can be a source for microbial growth due to its composition rich in macro and micronutrients and exposure to room temperature, factors that are conducive to microbial multiplication and increase the risk of hospital-acquired infections (Martins *et al.*, 2007; Pinto *et al.*, 2004).

However, it is recognized that the microbiological control of enteral diets is difficult because the meals need to be consumed immediately, not allowing a previous analysis. Therefore, it is necessary to implement a control system of critical points and a strict adoption of good practices in the preparation of enteral diets, with continuous training of the staff in order to prevent contamination (Pinto *et al.*, 2004; Simon *et al.*, 2007). According to the RDC nº 63 of 2000 of the National Health Surveillance Agency (ANVISA) (Brasil, 2000), it is a formal and mandatory condition the constitution of a Multidisciplinary Team in Nutritional Therapy (MTNT), responsible for ensuring appropriate conditions in all stages of ENT in order to obtain the best benefits of the procedure and prevent risks of contamination. This team is also responsible for training the staff involved in the preparation and administration of enteral feeding.

Mehall *et al.* (2002) highlight that actual contamination of enteral formulas and consequences to the body are unknown. These authors found in their study a relation between food intolerance and less weight gain in newborns fed with contaminated enteral tubes. Other authors also found an association between contaminated enteral diets and a higher incidence of diarrhea in patients (Okuma *et al.*, 2000). Therefore, delivery of enteral diets must assure satisfactory health hygienic conditions to allow a satisfactory clinical evolution of the patients.

A study that sought to assess the microbiological quality handmade enteral diets, prepared in households, observed a reduction in the counts of microorganisms that indicated a pattern of poor hygiene, with the correction of inappropriate handling practices. However as the contamination of these diets was still considered a problem by these authors, they suggested the creation of an instrument that would be able to investigate better the causes of this type of food contamination (Santos *et al.*, 2013).

Thus, it is clear the importance of assessing and monitoring possible contamination sources involved in the preparation and handling of enteral formulas, with emphasis on the surfaces of utensils and equipment, food handlers, and ingredients used in the preparation (water, supplements, and *in natura* foods). Control can be achieved by adopting sanitation and hygiene practices for the environment, foods, and hands of handlers. The latter is of crucial importance, because Enteral Nutrition (EN) handling at the bedside is a critical point for meals contamination by pathogenic bacteria (Okuma *et al.*, 2000; Lima *et al.*, 2005; Roy *et al.*, 2005; Maurício *et al.*, 2008).

This study was conducted with the purpose of evaluating the microbiological quality of non-industrialized (so-called "homemade" or "handmade" diets prepared in the healthcare premises) and industrialized diets, and the aseptic conditions of preparation and use of such feeds, produced in a hospital in the Jequitinhonha Valley, Minas Gerais, Brazil.

## Material and Methods

This study was conducted in a hospital in the Jequitinhonha Valley, Brazil, Minas Gerais and in the Laboratory of Food Hygiene of the Federal University of Jequitinhonha and Mucuri Valleys (UFVJM), Brazil, from July 2010 to November 2011.

### Collection of samples of enteral diets

Twenty-five samples of enteral diets were analyzed, 13 of them being non-industrialized diets (prepared in the hospital facility) and 12 industrialized diets, collected in two different times, immediately after the completion of preparation (T0) and after administration to the patient (T1), totalizing 50 sample units, each of them containing at least 100 mL of the diet. The "handmade" preparations consisted of: UHT (or long-life) whole milk, protein supplement (Nutren Active^®^, Nestlé, Brazil), pureed fruits, and vegetable soup (potato, chayote, carrot, and ground beef). The industrialized diets were of the type Isosource 1.5 cal^®^ and Fibersource 1.2 Kcal^®^. Samples from T0 were placed into previously sterilized plastic containers, and T1 samples were collected directly from the original containers of enteral diets. Then, the samples were transported in styrofoam boxes under refrigeration and analyzed not later than 6 h after being collected.

### Samples collection from surfaces and water

Twenty-seven samples were taken from surfaces and water: 6 swab samples of the countertop where foods are prepared, 6 swabs of the measuring cup, 11 swabs of EN administration devices, and 4 samples of the water used to dilute preparations and flush out the catheter. Swab samples were taken using tubes containing 10 mL water of saline solution (NaCl 0.85%). In a recipient previously sterilized in an autoclave, 250 mL of water were collected.

### Microbiological analysis

The diets were analyzed for total coliforms (35 °C) and thermo-tolerant coliforms (45 °C), *Escherichia coli*, viable aerobic mesophilic bacteria, *Salmonella* spp., *Listeria* spp*.*, *Listeria monocytogenes*, coagulase-positive *Staphylococcus*, and *Pseudomonas* spp. For countertops and utensils, total and thermo-tolerant coliforms, *E. coli*, *S. aureus*, coagulase-positive *Staphylococcus,* and viable aerobic mesophilic bacteria were analyzed. Analyses of water and EN administration devices were conducted to detect *Pseudomonas* spp. besides the microorganisms cited for evaluation of countertops and utensils.

### Samples dilution and homogenization

Twenty-five grams of each sample were weighed and homogenized in a Stomarc samples homogenizer model MA440/CF (Marconi^®^), in 225 mL of water saline solution (NaCl 0.85%). From this homogenate, decimal dilutions of up to 10^−6^ were made for the handmade diets and 10^−3^ for the industrialized diets and water, using tubes with 9 mL of water saline solution (NaCl 0.85%). The analyses of the samples of countertops, utensils, and administration devices were conducted in tubes containing 10 mL of this same solution (NaCl 0.85%).

### Counts of total and thermo-tolerant coliforms, and *E. coli*

One mL of each dilution was transferred to three test tubes containing Lauryl Sulfate Tryptose (LST) (HiMedia, USA) with Durhan tubes in its interior. It was used the Brilliant Green Bile Broth 2% (HiMedia, USA) for confirmation of total coliforms and *E. coli* broth (EC) (Acumedia, USA) for detection of thermo-tolerant coliforms. In addition to the Most Probable Number (MPN) method, was used Petrifilm EC plates (3M Microbiology, St. Paul, MN, USA) for the detection of coliforms and *E. coli.*

For total coliforms and *E. coli* incubation was performed at 35 °C for 24–48 h. The analyses of thermo-tolerant coliforms followed incubation at 45 °C for 24 h.

The results were expressed in Colony-Forming Units per milliliter of diet or water (cfu/mL), Most Probable Number per milliliter of diet or water (MPN/mL), cfu/cm^2^ of surface and cfu/administration devices.

### Counts of viable aerobic mesophilic bacteria

One mL of the dilutions described above was transferred to a Petri plate by the pour plate method, to which standard Plate Count Agar (HiMedia, USA) was added. After the agar homogenization and solidification, the plates were incubated at 35 °C for 24–48 h. The results were expressed in cfu/mL of diet or water, cfu/cm^2^ of surface and cfu/administration equipment.

### Detection of *Listeria* spp. and *L. monocytogenes*

The analysis of *L. monocytogenes* was initially performed by adding 225 mL of Listeria Enrichment Broth (M569, Himedia, USA) in 25 g of sample, followed with incubation at 35 °C for 24 h. Immediatly after, it was made an inoculation of 0.1 mL of the previous step in tubes containing 10 mL of Fraser broth (Acumedia, USA), and incubated at 35 °C for 48 h. Furthermore, 0.1 mL of the same sample incubated for 24 h in Listeria Enrichment Broth was transferred to Oxford agar plates (Himedia, USA) and 0.1 mL to chromogenic agar plates (BioCen, Brazil), and incubated at 35 °C for 24–48 h, and checked for growth of typical colonies. With the tubes with Broth Fraser it was made the same plating mentioned, in Oxford agar plates and chromogenic agar plates, to check for growth of *L. monocytogenes* bacteria. In addition, analysis to detect *Listeria* spp. was conducted, using the kit TECRA *Listeria* VIA, AOAC 995.22, 2002.09 (AOAC, 2005) (3M Microbiology, St. Paul, USA). The results were expressed as presence or absence of *Listeria* spp. and *L. monocytogenes* in 25 mL of diet.

### Detection of *Salmonella* spp

Detection of *Salmonella* was performed using the pre-enrichment medium of 25 mL of samples diluted in 225 mL of Buffered Peptone Water (Himedia, USA), incubated at 35 °C for 22–24 h. After this period, the primary selective enrichment was accomplished: 1.0 mL of the pre-enrichment medium was transferred to tubes containing 9 mL of Selenite Cystine broth (Acumedia, USA), and incubated at 35 °C for 6–18 h. Next, the secondary selective enrichment was performed: 1 mL of the primary enrichment medium was transferred to tubes containing 9 mL of M broth (Himedia, USA), and incubated at 35 °C for 6–8 h. After that, the kit TECRA *Salmonella* VIA, AOAC 989.14, 998.09 (AOAC, 2005) (3M Microbiology, St. Paul, USA) was used. The results were expressed as presence or absence of *Salmonella* spp. in 25 mL of diet.

### Enumeration of coagulase-positive *Staphylococcus*

The analysis for *Staphylococcus* was carried out by direct count on plates using dilutions up to 10^−3^. One mL was transferred to 6 Petri plates with Baird-Parker agar (Himedia), 4 related to 10^−1^ dilution and two to 10^−2^ and 10^−3^ dilutions, and incubated at 35 °C for 48 h. After checking for typical colonies growth (black or gray, with two halos, one being opaque and the other translucent), count and selection of five typical colonies were performed for the coagulase test. The result was expressed in cfu/mL of diet.

In the analyses of countertops, utensils, water, and administration devices, for the counts of *Staphylococcus* Petrifilm™ Staph Express plates (3M Microbiology, St. Paul, MN) were used, and after growth of the colonies (purple or black with halo), the referred coagulase test was carried out.

### Counts of *Pseudomonas* spp

To count *Pseudomonas* spp. the same dilutions, as already described, were used, up to 10^−3^, and then 0.1 mL was poured onto Petri plates with *Pseudomonas* agar for Piocianines (M119, Himedia, USA), and then incubated at 25 °C for 48 h. As colonies grew (white, round shaped), a confirmation test was performed in inclined tubes with Triple Sugar Iron - TSI (Himedia, USA) and then incubated at 25 °C for 24 h. The isolates growing without change in color were confirmed as *Pseudomonas* spp.

### Administration of questionnaire

The assessment of good practices of diets preparation and handling was according to the Brazilian technical regulation for enteral nutrition therapy(Brasil, 2000).

This regulation contains guidelines for inspection of EN-related activities, including the following: MTNT activities, general conditions, receiving of diet prescriptions, storage, water, preparation, cleaning and sanitation, clothing, handling and packaging, storage and transport, quality assurance, quality control, and EN administration.

Each item had questions, classified as essential (E), when it would impact critically the EN quality and safety; necessary (N), which would impact less critically the EN quality and safety; and recommendable (R), which would not interfere critically on the EN safety and quality.

For the interpretation of the results, each question was considered "conform" (C), when it met the requirements; "nonconform", when the item being investigated did not comply with the requirements; and "not applicable" (NA), when the hospital did not provide the service.

### Statistical analysis

The results were analyzed by means of the difference between the values of medians using Mann Whitney and Wilcoxon Signed Rank nonparametric testing, correlation and regression analysis using the Minitab software, version 15. The statistical significance level was 5% of probability.

## Results and Discussion

Both industrialized and non-industrialized enteral diets showed contamination by coliforms and thermo-tolerant coliforms, aerobic mesophilic bacteria, and *Pseudomonas* spp.

Considering the microbiological standards under Brazilian law (Brazil, 2000), the percentage of inadequate samples was apparently high, especially in handmade diets and second collection time (T1) (Table 1).

**Table 1:** Percentage of adequacy (yes) and inadequacy (no) with the microbiological standards of the Brazilian legislation, in handmade diets (HD) and industrialized diets (ID) in both moments (T0/T1), Diamantina, MG, 2013.

Microorganism (* Microbiological Standard)	HD		ID	
		
	yes (%) T0/T1	no (%) T0/T1	yes (%) T0/T1	no (%) T0/T1
aerobic mesophilic (< 10^3^ cfu/mL)	31/8	69/92	91.5/75	8.5/25
Total coliforms (< 3 cfu/mL)	46/0	54/100	100/83	0/17
thermo-tolerant coliforms (< 3 MPN/mL)	92/92	8/8	100/83	0/17

* Microbiological Standard according RDC nº 63 de 2000.

T0: Sample collected immediately after handling; T1: sample collected after being administered to the patient

These results corroborate those found by Oliveira *et al.* (2000), Lima *et al.* (2005), Maurício *et al.* (2008), Santos *et al.* (2013), and Borges *et al.* (2011). High counts of these microorganisms indicate poor hygiene and sanitation conditions, pointing to failures in the handling process, hygiene of equipment and utensils, or even hygiene of food handlers. It is also noteworthy that although the presence of such microorganisms does not necessarily indicate contamination by pathogens, it is a matter of concern because this kind of diet is delivered to individuals with reduced defenses, who are more susceptible to infections (Lima *et al.*, 2005).

The median counts of total coliforms and aerobic mesophilic was high both for handmade enteral diets as for industrialized, in the two moments of collection, after preparation (T0) and after administration to the patient (T1), being considered significant results (p < 0.05). No difference was observed in the counts of fecal coliform (T0 and T1) and *Pseudomonas* spp. (T1) (Table 2).

**Table 2:** Comparison between medians of counts of aerobic mesophilic microorganisms, total coliforms, thermo-tolerant coliforms, and *Pseudomonas* spp. between both types of diets (handmade and industrialized), according to the moment of collection (T0/T1) of enteral diets in a hospital in Diamantina, Minas Gerais, 2011.

Collection time	Microorganisms	Types of Diets	n	Median	p value
1^st^ moment (T0)	Total coliforms log (cfu/mL)	Handmade	13	1.301	0.005
		Industrialized	12	0.000	
	Thermo-tolerant coliforms log (MPN/mL)	Handmade	13	0.000	0.338
		Industrialized	12	0.000	
	Aerobic mesophilic microorganisms log (cfu/mL)	Handmade	13	3.342	< 0.001
		Industrialized	12	0.000	
	*Pseudomonas* spp. log (cfu/mL)	Handmade	13	2.000	0.004
		Industrialized	12	0.000	
2^nd^ moment (T1)	Total coliforms log (cfu/mL)	Handmade	13	1.903	0.006
		Industrialized	12	0.000	
	Thermo-tolerant coliforms log (MPN/mL)	Handmade	13	0.000	0.636
		Industrialized	12	0.000	
	Aerobic mesophilic microorganisms log (cfu/mL)	Handmade	13	3.633	0.002
		Industrialized	12	1.952	
	*Pseudomonas* spp. log (cfu/mL)	Handmade	13	2.556	0.095
		Industrialized	12	0.000	

Mann Whitney Test. Significance level: p < 0.05.

**Figure 1:**
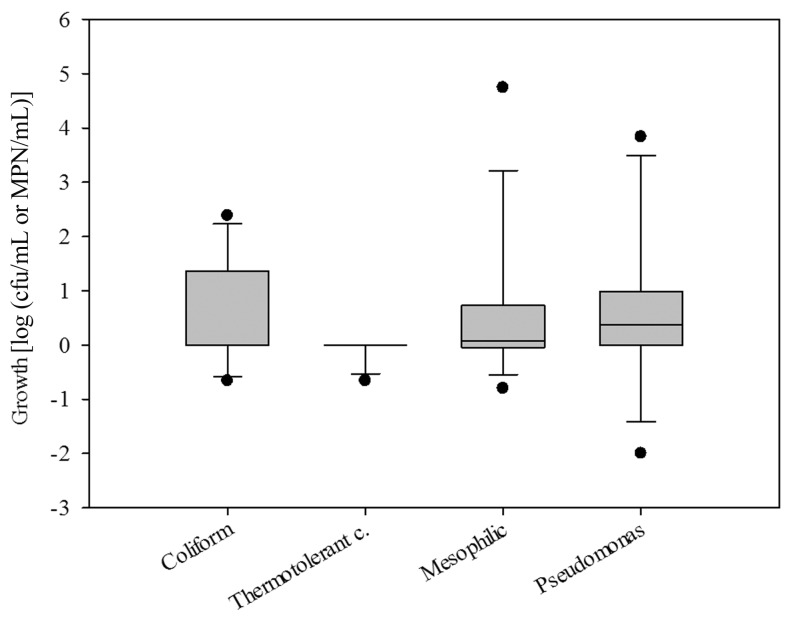
Increased amount of microorganisms found between the two moments of collection, after preparation (T0) and after administration (T1) (Wilcoxon Signed Rank test) of handmade and industrialized enteral diets served in a hospital of the Valley of Jequitinhonha - Minas Gerais, 2011.

Taking into account the difference between the two instants of collection (T0 and T1), regardless of the type of diet, an increased number of microorganisms (Figure 1) were found, and such growth was statistically significant (p < 0.05), except for the counts of thermo-tolerant coliforms (p = 0.500).

There was a significant correlation (p < 0.05) between the time elapsed from the preparation of the diet until completion of administration to the patients and the increased population of *Pseudomonas* spp., not significant for the counts of other bacteria analyzed (p > 0.05). The regression equation that best represents the increased amount of *Pseudomonas* spp. as a function of time is:

log (cfu/mL)=0.0158*time.

So, we can infer that at every minute there is an increase of 0.0158 log (cfu/mL) of *Pseudomonas* spp. population (p = 0.017).

With regard to contamination by *Pseudomonas* spp., it is worth noting that the Brazilian legislation does not specify standards for this pathogen, but it is important to consider that its occurrence has been often observed in nosocomial infections or in isolated clinical materials sent to culture (Villas Bôas and Ruiz, 2004; Banderó Filho *et al.*, 2006), which makes the result found in the present study relevant.

Coagulase-positive *Staphylococcus*, *Salmonella* spp., and *L. monocytogenes* counts were not detected. However, a sample of a milk-based handmade diet was positive for *Listeria* spp.

In a study conducted by Pinto *et al.* (2004), the presence of pathogens like *L. monocytogenes* in this kind of diet was related to those that had greater manipulation and a large variety of foods. Therefore, this kind of contamination can refer either to the type of product used or to inadequate preparation and handling techniques.

For the analyses of the surfaces and utensils used in the enteral feeding preparation, counts up to 64.8 cfu/cm^2^ of mesophilic microorganisms, 6 cfu/cm^2^ of coliforms, and 0.3 cfu/cm^2^ of *S. aureus* were found. Regarding the utensil investigated, it was observed mesophilic bacteria counts of up to 10^5^ cfu/utensil, coliforms present in values of 5.3 × 10^4^ cfu/utensil, and 2 × 10 cfu/utensil of *S. aureus*.

For the analysis of surfaces and utensils used in handling enteral feeding there are no standards in legislation regarding their microbiologic quality. Martins *et al.* (2007) used a recommendation by APHA of 2 cfu/cm^2^ for aerobic mesophils. In the present work, using the same reference, among the six samples of countertops analyzed, three did not show satisfactory results for aerobic mesophils and one for coliforms, indicating inefficient hygiene procedures. Regarding the utensil investigated, the samples were inadequate as to counts of mesophils, coliforms, and *S. aureus*. Carvalho Filho *et al.* (2008) and Borges *et al.* (2011) found results that corroborate our study. The authors pointed out that inadequate structure and hygiene can contribute to contamination of diets and emphasized that the best practices for preparation provides that equipment and utensils should not be a source of contamination for enteral diets.

With respect to the water samples used to cleanse the tubes and to dilute the handmade diets, it was observed absence of total coliforms and *Pseudomonas* spp. and counts of mesophilic microorganisms of up to 4.6 × 10 cfu/mL and coagulase-negative *S. aureus* of 8.5 × 10 cfu/mL. Regarding the analysis of the EN administration devices, two samples showed contamination by coliforms (up to 10^4^ cfu/device), *E. coli* (up to 10^2^ cfu/device), aerobic mesophilic microorganisms (up to 10^4^ cfu/device), coagulase-positive *Staphylococcus* (10^2^ cfu/device).

With respect to the water quality, we considered that it is not a source of contamination, once it met the standards defined in the RDC N°. 12, of January 12, 2001 (ANVISA, 2001).

The contamination of catheters was considered an important risk factor for contamination of enteral feeding, including by pathogens, allowing the occurrence of infectious exacerbations in patients receiving this type of nutritional support. Mehall *et al.* (2002) found 94% of contamination in EN tubes used in newborns. These authors suggest that the organisms found mirror the nurses' hands, and this requires special attention. According to Luft *et al.* (2006), nonfulfillment of standard operational procedures in EN administration may be a key factor for the increased incidence of diarrhea in patients receiving this kind of feeding.

### Good practices questionnaire

**Figure 2:**
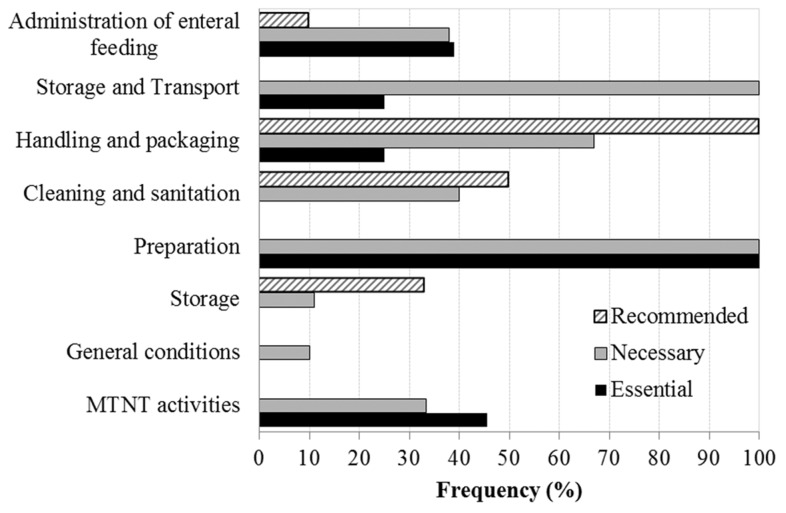
Percent distribution of nonconformities among essential, necessary, and recommended items per stage of production and administration of enteral feeding in a hospital in the Valley of Jequitinhonha, Minas Gerais, 2011.

The questionnaire included 208 topics for investigation, and Figure 2 represents the percentage of nonconformities found for each classification: essential, necessary or recommended.

The following topics did not show nonconformities: receiving of the diet prescription, water, and clothing.

Among the questions related to MTNT activities, the hospital in question succeeded in some topics considered essential, such as the existence of a formal act to constitute the team, formal record of meetings, and records of medical prescriptions. However, the following were considered nonconformities: lack of procedural protocols for the professionals involved in the therapy, lack of training programs, and lack of ENT quality indicators.

Regarding the activities related to preparation, general conditions, cleaning and sanitation, handling, storage, packaging and transport, the main nonconformity found in the category of essential was the relative lack of a separate area for preparation of EN. The other failures detected were among items considered necessary and recommended, and among them we can cite: lack of sealed openings and wire-protected windows, allowing access of insects and rodents into the EN handling sector, unrestrictive circulation of people in the preparation sector, and the floor of the preparation area was difficult to clean and had cracks.

Among the conformities found for all categories (essential, necessary and recommended), it is worth noting that preparation was made only upon medical prescription, the staff was trained to perform this activity and such training was recorded, the diets were kept in an exclusive refrigerator, and the diets production facility of this healthcare institution performs sanitation and pest control every semester.

Concerning the enteral feeding process, the nonconformities found among the items indicated as essential include: the diets were not stored in the refrigerator when not used at the specified time; the feeding devices were not exclusively used for feeding, being also used for administration of medications; sanitation of tube connections was not performed during the change of these devices.

Among the items considered essential, the conformities found among the EN administration activities, we can cite the clinical and laboratorial control of the patient receiving the enteral diet, and the diet was administered in its original container. In addition, among the topics considered necessary and recommended, we can cite pump-assisted administration of the diet; trained nursing staff to use the pumps; and existence of a manual of procedures for EN administration.

Situations not applied to the reality of this healthcare unit are mainly related to the fact that MTNT has not established Standard Operational Procedures (SOP) and therefore there are no control records of essential qualities to assure EN safety.

Maurício *et al.* (2008) used the same instrument of assessment of good practices as used in the present work and found nonconformities similar to those detected in this study, such as a separate area exclusively for handling enteral diets and structural problems. These authors also emphasize that noncompliance of an item classified as essential would be cause of immediate suspension of EN diets production. Therefore, this healthcare unit needs to implement a strict program of goods practices for the preparation and handling of enteral diets and an efficient system of quality control to be developed by the MTNT.

## Conclusions

The microbiological quality of the enteral diets analyzed was not satisfactory, and the aseptic conditions in the investigated hospital with respect to preparation and handling of enteral diets were favorable to risks of cross contamination.
